# An integrated neuroimaging-omics approach for the gut-brain communication pathways in Alzheimer’s disease

**DOI:** 10.3389/fnagi.2023.1211979

**Published:** 2023-10-06

**Authors:** Can Sheng, Wenying Du, Yuan Liang, Peng Xu, Qingqing Ding, Xue Chen, Shulei Jia, Xiaoni Wang

**Affiliations:** ^1^Department of Neurology, The Affiliated Hospital of Jining Medical University, Jining, China; ^2^Department of Neurology, China-Japan Friendship Hospital, Beijing, China; ^3^Institute of Microbiology, Chinese Academy of Sciences, Beijing, China; ^4^Department of Neurology, Sir Run Run Shaw Hospital, School of Medicine, Zhejiang University, Hangzhou, China

**Keywords:** Alzheimer’s disease, microbiome, metabolome, neuroimaging, microbiota-gut-brain axis

## Abstract

A key role of the gut microbiota in the pathogenesis of neurodegenerative diseases, such as Alzheimer’s disease (AD), has been identified over the past decades. Increasing clinical and preclinical evidence implicates that there is bidirectional communication between the gut microbiota and the central nervous system (CNS), which is also known as the microbiota-gut-brain axis. Nevertheless, current knowledge on the interplay between gut microbiota and the brain remains largely unclear. One of the primary mediating factors by which the gut microbiota interacts with the host is peripheral metabolites, including blood or gut-derived metabolites. However, mechanistic knowledge about the effect of the microbiome and metabolome signaling on the brain is limited. Neuroimaging techniques, such as multi-modal magnetic resonance imaging (MRI), and fluorodeoxyglucose-positron emission tomography (FDG-PET), have the potential to directly elucidate brain structural and functional changes corresponding with alterations of the gut microbiota and peripheral metabolites *in vivo*. Employing a combination of gut microbiota, metabolome, and advanced neuroimaging techniques provides a future perspective in illustrating the microbiota-gut-brain pathway and further unveiling potential therapeutic targets for AD treatments.

## 1. Introduction

Alzheimer’s disease (AD) is the most common form of dementia, leading to a globally heavy healthcare and economic burden (Jia et al., 2018, 2020). According to the National Institute on Aging and Alzheimer’s Association (NIA-AA) research framework, AD-related biomarkers are mainly classified into three existing groups, including amyloid-β (Aβ) deposition, pathologic tau aggregation, and neurodegeneration [AT(N)] (Jack et al., 2018; Scheltens et al., 2021). However, the failure or unsatisfactory results of recent clinical trials targeting Aβ clearance underscores the importance of understanding new disease-driving mechanisms and seeking other available disease-modifying therapies.

Gut microbial dysbiosis may serve as a key factor for developing AD (Cryan et al., 2020). A large number of studies have presented compelling evidence that there is bidirectional communication between the gut microbiota and brain (Cryan et al., 2019). Indeed, alterations in the composition and function of the gut microbiota have been reported both in human AD patients (Vogt et al., 2017; Zhuang et al., 2018; Li et al., 2019; Liu et al., 2019b; Sheng et al., 2021, 2022) and mouse models of AD (Zhang et al., 2017; Chen et al., 2020; Kim et al., 2020; Chen et al., 2022). However, knowledge about underlying mechanisms of the gut microbiota in regulating the brain of AD remain unclear. A growing evidence supports that AD appears to be a metabolic disease (Kaddurah-Daouk et al., 2013; Varma et al., 2018; Whiley et al., 2021). One of the primary mediating pathways by which the gut microbiota interacts with the host brain is *via* peripheral metabolites, which are small molecules that are derived from bacterial metabolism of the non-digestible carbohydrates, the products of cholesterol metabolism, or directly from gastrointestinal bacteria, *etc* (Luan et al., 2019; Lavelle and Sokol, 2020). Many metabolites play a series of roles in maintaining host health, including brain energy homeostasis, gut mucosal integrity, the host immune system and membrane lipid metabolism (Lavelle and Sokol, 2020). Gut microbiota can also give rise to metabolites, such as tryptophan derivatives and short-chain fatty acids (SCFAs), which may play an important role in modulating cognitive function and behavior (Silva et al., 2020; Doifode et al., 2021). Recently, advanced metabolomics techniques have been used to identify key AD-related metabolites and define changed metabolic pathways during AD disease trajectory (Toledo et al., 2017; Sun et al., 2020). Therefore, the integration of microbiome and metabolomics can help reveal the potential impact of altered microbial communities on host metabolism. Nevertheless, how do gut microbiome and peripheral metabolism impact brain health, and further cognitive function?

Interestingly, the advances in neuroimaging techniques may provide a promising approach to directly mirror the impact of the gut microbiota on human brain *via* the mediation role of peripheral metabolites (Cryan et al., 2019; Liu et al., 2019a; Arnoriaga-Rodríguez et al., 2020). Multi-modal magnetic resonance imaging (MRI), including structural MRI (sMRI), functional MRI (fMRI) and diffusion tensor imaging (DTI), as well as [^18^F] fluorodeoxyglucose-positron emission tomography (FDG-PET), have offered an avenue that allows non-invasively investigating abnormalities of brain structure, spontaneous functional activities and glucose metabolism in AD (Wang et al., 2020). Therefore, the combination of neuroimaging, microbiome and metabolomics, which is also called neuroimaging-omics, has the potential to identify multi-dimensional biological signatures essential to reveal the complex gut microbiota-metabolites-brain interaction mechanism.

In this review, we aim to summarize: (1) the microbiota-gut-brain axis and AD; (2) the potential mediator of the gut microbiota-brain pathway: peripheral (blood and gut) metabolites; (3) the integrative neuroimaging-omics method for studying AD. This review will facilitate a better understanding of the interplay between microbiota, metabolites and neurodegeneration along the gastrointestinal-brain axis in AD, possibly providing future targets for AD precision therapeutic modulation (see Table 1).

**Table 1:** Summary of neuroimaging-omics studies in human AD.

**Study**	Participants	Study design	Multi-omics techniques	Main findings
Liang et al. (2022)	Discovery cohort (*n* = 1,430) Replication 1 (AD 30, MCI 30, NC 30) Replication 2 (*n* = 1,300)	Cross-sectional study	(1) Structural MRI (2) 16S rRNA+Metagenomics (3) Targeted metabolomics	*Odoribacter* was positively associated with hippocampal volume, which might be mediated by acetic acids.
Asaoka et al. (2022)	MCI (*n* = 130)	RCT	(1) Structural MRI (2) 16S rRNA	Probiotics *Bifidobacterium breve* consumption for 24 weeks suppressed brain atrophy progression in MCI patients.
Verhaar et al. (2021)	AD (*n* = 33) MCI (*n* = 21) NC (*n* = 116)	Cross-sectional study	(1) Structural MRI (2) 16S rRNA	*Lachnospiraceae NK4A136 group* spp. and *Anaerostipes* spp. correlated with lower global cortical atrophy visual scores on MRI.
Liu et al. (2021)	MCI (*n* = 20) NC (*n* = 22)	Cross-sectional study	(1) Resting-state functional MRI (2) 16S rRNA	In aMCI, at the typical band, *Bacteroides* was negatively correlated with fALFF of cerebellar vermis IV-V, and the *Ruminococcaceae* was negatively correlated with fALFF of left lenticular nucleus and pallidum. The *Clostridiaceae* and *Blautia* were positively correlated with the left cerebellum lobules IV-V at the slow-4 band. The *Veillonellaceae* was positively correlated with fALFF of left precentral gyrus at the slow-5 band.
Saji et al., (2019)	MCI (*n* = 61) NC (*n* = 21)	Cross-sectional study	(1) Structural MRI (2) 16S rRNA	MCI patients with more *Bacteroides* were more likely to present with white matter hyperintensity and cortical and hippocampal atrophy.
Nho et al. (2019)	AD (*n* = 305) LMCI (*n* = 505) EMCI (*n* = 284) SMC (*n* = 98) NC (*n* = 370)	Cross-sectional study	(1) Structural MRI (2) [^18^F] FDG-PET (3) Targeted metabolomics	Bile acids levels were associated with hippocampal volume, cortical thickness, and brain glucose metabolism.
Toledo et al. (2017)	AD (*n* = 175) MCI (*n* = 356) NC (*n* = 199)	Longitudinal study	(1) Structural MRI (2) Targeted metabolomics	Lower valine and α–AAA values were associated with faster cognitive decline, similarly the coefficient for valine was negatively associated with ventricular volume changes.
Arnold et al. (2020)	AD (*n* = 302) EMCI (*n* = 270) MCI (*n* = 490) SMC (*n* = 93) NC (*n* = 362)	Cross-sectional study	(1) [^18^F] FDG-PET (2) Targeted metabolomics	There was negative association between C16:1 and FDG-PET, especially in male group.
Simpson et al. (2016)	Baseline: NC (*n* = 107) Follow-up: AD (*n* = 16) MCI (*n* = 7)	Longitudinal study	(1) PET (2) Metabolomics	Lower plasma PCs concentrations were associated with lower rCBF in several brain regions, including bilateral frontal lobe, right superior temporal gyrus, left middle temporal gyrus, left anterior cingulate cortex, etc.

AD, Alzheimer’s disease; MCI, mild cognitive impairment; NC, normal control; EMCI, early mild cognitive impairment; LMCI, late mild cognitive impairment; SMC, subjective memory complaint; RCT, randomized controlled trial; MRI, magnetic resonance imaging; rRNA, ribosomal RNA; [18F] FDG-PET, [18F] fluorodeoxyglucose-positron emission tomography; fALFF, fractional amplitude of low-frequency fluctuation; α-AAA, α-aminoadipic acid; rCBF, regional resting state cerebral blood flow; PC, phosphatidylcholines.

## 2. Microbiota-gut-brain axis and AD

Accumulating evidence suggests a close correlation between altered gut microbiota and brain, which is also called the microbiota-gut-brain axis. Alterations of the gut microbiota are associated with abnormal brain Aβ deposition in both AD animal models (Chen et al., 2020, 2022) and humans (Cattaneo et al., 2017; Sheng et al., 2022), and the transfer of a healthy fecal microbiota reduces Aβ and tau pathology in AD animal models (Kim et al., 2020). Gut microbiota-host interactions may lead to the release of numerous substances, such as cytokines, neurotransmitters, neuropeptides, and gut-derived metabolites, into the blood and lymphatic system. These substances can permeate into the brain through the blood brain barrier (BBB), or directly influence the neural messages transmission carried by the vagal and spinal afferent neurons (Cryan et al., 2019), which may further regulate brain function and behavior. Thus, targeting gut microbiota would provide a critical opportunity for therapeutic intervention of AD.

Recently, cross-sectional preclinical and clinical studies have revealed the significantly altered gut microbiota in AD. Compared with cognitively normal individuals, patients with AD and MCI showed significantly decreased microbial diversity and changed relative abundance of phylum *Firmicutes* and its corresponding bacteria (Vogt et al., 2017; Sheng et al., 2021, 2022). Similarly, amnestic mild cognitive impairment (MCI) patients also exhibited significant alterations in the microbial compositions, such as *Gammaproteobacteria*, *Enterobacteriales* and *Enterobacteriaceae*, the relative abundance of which were between cognitively normal adults and AD patients (Liu et al., 2019b). Additionally, one recent study reported the decreased anti-inflammatory genus *Faecalibacterium* in individuals with subjective cognitive decline (SCD), providing the preliminary evidence of altered gut microbiota in elderly adults at risk of AD (Sheng et al., 2021). For Aβ positive cognitively normal individuals, the relative abundance of phylum *Bacteroidetes* was enriched, whereas taxa in *Firmicutes* and *Proteobacteria* phyla were reduced (Sheng et al., 2022). Besides, the global brain Aβ burden was negatively associated with the *Desulfovibrionaceae*, *Bilophila* and *Faecalibacterium*, which confirmed the association of gut microbial dysbiosis with AD. Thus, gut microbiota may serve as a potential peripheral biomarker in identifying AD. Nevertheless, the regulatory mechanism of gut microbiota on the brain is unclear.

## 3. The potential mediator of the gut microbiota-brain pathway: peripheral metabolites

Currently, there is increasing interest in the interaction of gut microbiota with the brain *in vivo*. Metabolomics may provide an approach to understand why specific strains/probiotic interventions have the potential to modulate cognitive function. Metabolomics is an omics analysis that measures thousands of small molecule metabolites simultaneously (Luan et al., 2019; Lavelle and Sokol, 2020). Advances in high-throughput metabolomics techniques have provided new biochemical insights into disease mechanisms and revealed early systemic changes in disease. Given that the process of AD includes many biochemical changes, such as phosphorylation of tau, oxidative stress, and membrane lipid dysregulation (Toledo et al., 2017), the application of metabolomics may offer a promising perspective in AD diagnosis and functional pathway elucidation.

AD is now considered a metabolic dysfunction disease, with significant peripheral metabolic disturbances (Toledo et al., 2017; Varma et al., 2018; MahmoudianDehkordi et al., 2019; Arnold et al., 2020; Huo et al., 2020). Blood- and gut microbiota-derived metabolites are regarded as the potential peripheral metabolites, which may play the important role in microbiota-host crosstalk in AD. Lipid metabolism dysfunction is a hallmark in the whole spectrum of AD, with the declined level of fasting serum sphingomyelin (SM) and ether-containing phosphatidylcholines (PC) in the asymptomatic stage of AD, as well as the decreased acylcarnitines, valine and α-aminoadipic acid (α-AAA) mainly in the symptomatic stage of AD (Toledo et al., 2017). To our knowledge, ether-linked PCs and SMs are rich in the lipid rafts of the membrane. Lipid rafts are fluctuating assemblies composed of sphingolipid, cholesterol and proteins, forming platforms for membrane signaling and trafficking (Lingwood and Simons, 2010). In addition, accumulating evidence suggests that lipid rafts act as a common platform for the progression of AD pathology, which may be associated with amyloid precursor protein (APP) processing, Aβ accumulation and tau oligomers production (Rushworth and Hooper, 2010; Kawarabayashi et al., 2022). Therefore, changed lipid metabolism may provide a novel target for AD treatment. Moreover, current evidence from pre-clinical studies indicates that several lipid substances, such as bioactive sphingolipids, have the potential to modulate the gut microbiota and gut barrier function (Norris et al., 2016, 2019; Rohrhofer et al., 2021). For instance, intake of SMs shows protective properties against gut dysbiosis (Norris et al., 2019), indicating the interaction between gut microbiota and metabolites.

Short-chain fatty acids (SCFAs), as the most common form of gut microbiota-derived metabolites, also build a vital communication “bridge” between the gut and brain (Dalile et al., 2019; Colombo et al., 2021; Doifode et al., 2021; Ameen et al., 2022). About 90% SCFAs are derived from indigestible carbohydrates in the cecum and colon through gut microbial fermentation. SCFAs play a key role in the homeostasis of CNS by maintaining the integrity of intestinal epithelium barriers (Feng et al., 2018), regulating the metabolism of glucose and lipid (Ge et al., 2008; Canfora et al., 2015), and modifying immune function (Corrêa-Oliveira et al., 2016). Previous studies showed that the concentration of fecal SCFAs significantly declined in AD mice (Zhang et al., 2017). Wu et al. Wu et al. (2021) initially investigate the fecal metabolomics in the spectrum of AD, characterized by the progressively decreased trend of SCFAs from healthy controls to aMCI and AD patients. We speculate that the declined SCFAs level in AD may be due to the significant reduction of key SCFA-producing bacteria belonging to phylum *Firmicutes*, such as class *Clostridia*, order *Clostridiales*, family *Ruminococcaceae* and family *Lachnospiraceae* (Liu et al., 2019b; Sheng et al., 2021, 2022; Chen et al., 2023). Thus, SCFAs also exhibit the mediating impact between the gut microbiota and the brain.

## 4. An integrative neuroimaging-omics method for AD study

### 4.1. The framework of the neuroimaging-omics analysis

Neuroimaging techniques have the potential to reflect the microbiota-gut-brain interaction, and evaluate the effect of interventions targeting the gut microbiota (Tillisch et al., 2008; Tillisch and Labus, 2014). To date, several studies have explored the correlation between the gut and brain in other diseases using neuroimaging techniques (Wang et al., 2019; Arnoriaga-Rodríguez et al., 2020). For instance, a prior study has investigated the linkages of gut microbiota, systematic inflammation, amygdala-based functional connectivity, and cognitive performance in patients with end-stage renal disease. Causal mediation analysis revealed that the disrupted *Roseburia* indirectly regulated the amygdala-based functional connectivity through systematic tumor necrosis factor-alpha (Zheng et al., 2020). Tillisch and colleagues once reported that a four-week intake of a fermented milk product with probiotics by healthy women affected the activity of brain regions that control the central processing of emotion and sensation (Tillisch et al., 2013), providing direct evidence that intestinal bacteria can influence brain activity in healthy adults.

Currently, multimodal neuroimaging techniques, including sMRI, fMRI, DTI, and FDG-PET, can reveal subtle brain structural, functional, and glucose metabolism changes in AD (Wang et al., 2020). During the neuroimaging data preprocessing and analysis, multi-dimension features will be extracted, such as gray matter volume, amplitude of low frequency fluctuation (ALFF), fractional ALFF (fALFF), fractional anisotropy (FA), mean diffusivity (MD), etc. Using the radiomics method, high-throughput features (e.g., gray level histogram, texture, and wavelet transform) are also extracted from different brain tissues (Feng and Ding, 2020; Li et al., 2020), which will provide more precise evidence for the diagnosis of AD.

Advances in high-throughput sequencing have promoted the rapid development of microbiome research. Intestinal microbiome sequencing analyses mainly includes 16 ribosome DNA (rDNA) amplicon and metagenomic sequencing. 16S rDNA amplicon sequencing is the most commonly used method for neurological diseases. The advantages of 16S rDNA amplicon sequencing are cost effective, relatively small data amount, and quick analysis. However, it can only reach genus-level resolution, and it is sensitive to the specific primers and the number of PCR cycles (Liu et al., 2020). Metagenomic sequencing analysis, with higher taxonomic resolution (species- or strain-level) and potential functional information, will provide more microbial information than traditional 16S rDNA amplicon sequencing (Quince et al., 2017; Liu et al., 2020).

**Figure 1:**
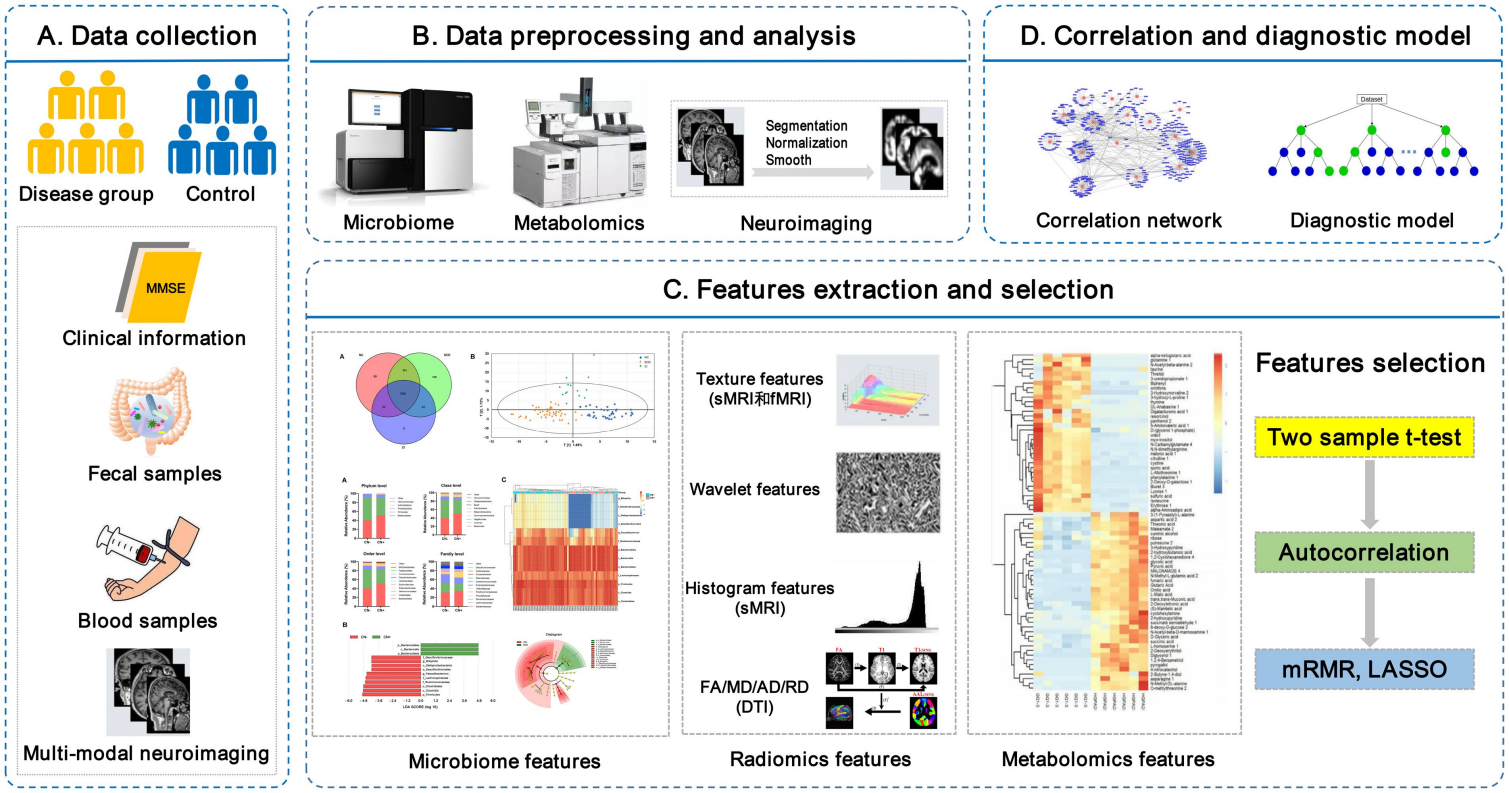
The framework of the neuroimaging-omics analysis. **(A)** Data collection: recruitment of the disease group and control group, collection of the clinical information, fecal samples, fasting blood samples, and multi-modal neuroimaging scan data. **(B)** Data preprocessing and analysis: including microbiome high-throughput sequencing, untargeted/targeted metabolomics, images segmentation, normalization, etc. **(C)** Features extraction and selection: key multi-omics features are extracted and further selected using some algorithms, such as Max-Relevance and Min-Redundancy (mRMR) and least absolute shrinkage and selection operator (LASSO). **(D)** Correlation and diagnostic model: the correlation network is used to investigate the complex relationship between different omics features, and the establishment of diagnostic models is based on the selected key multi-omics features. LASSO, least absolute shrinkage and selection operator; mRMR, Max-Relevance and Min-Redundancy; sMRI, structural MRI; fMRI, functional MRI; DTI, diffusion tensor imaging.

Peripheral metabolites (fecal and plasma/serum) exert diverse effects on host physiology and are likely to be the key mediators between the gut microbiota and brain. Thus, the combination of brain imaging techniques with microbiome and metabolomics will greatly enhance our understanding of the microbiota-gut-brain axis in regulating cognition in AD (Cryan et al., 2019). The proposed framework of the neuroimaging-omics analysis is shown in Figure 1.

### 4.2. Neuroimaging and microbiome in AD

De Santis has provided the definition of *radiomicrobiomics*, which is an approach designed to extract quantitative parameters by combining brain imaging and gut microbiome (e.g., gut microbial composition, alpha diversity) (De Santis et al., 2019), mining these big data to develop diagnostic biomarkers of diseases and further elucidate the causal nature of the gut-brain interaction. Accumulating evidence supports that alterations of the gut microbiome are associated with brain structural and functional changes. Using structural MRI and 16S rRNA analysis, researchers have confirmed that specific gut microbial features are associated with cognitive dysfunction and decreased hippocampal volume (Saji et al., 2019; Liang et al., 2022). A cross-sectional study including AD, MCI and cognitively normal individuals found that the higher abundance of *Lachnospiraceae NK4A136 group* spp. and *Anaerostipes* spp. was correlated with lower global cortical atrophy visual scores (Verhaar et al., 2021). In addition, MCI patients with more *Bacteroides* were more likely to present with cortical and hippocampal atrophy (Saji et al., 2019). Resting-state fMRI can also build the crosstalk between the gut microbiota and the human brain. For instance, in patients with aMCI, gut microbiota, such as *Bacteroides*, *Clostridiaceae*, and *Blautia*, was confirmed to interact with intrinsic brain activity at different bands (Liu et al., 2021). Moreover, one study has investigated the effect of the probiotic strain *Bifidobacterium breve MCC1274 (A1)* on preventing brain atrophy in patients with MCI (Asaoka et al., 2022). The results showed that probiotics *Bifidobacterium breve* consumption for 24 weeks suppressed brain atrophy progression in MCI patients, validating that manipulating gut microbial compositions can ameliorate the pathogenesis of AD.

### 4.3. Neuroimaging and metabolomics in AD

An increasing amount of findings *in vivo* suggest that alterations of blood- and gut-derived metabolites, such as lipid metabolites, amino acids, bile acids, and SCFAs, may be closely linked to neuroimaging biomarkers of AD. Using targeted metabolomics, the declined concentrations of sphingomyelin (SM) and ether-containing phosphatidylcholines (PC) were associated with brain atrophy, suggesting that the dysfunction in lipid metabolism was linked to the brain morphological changes (Toledo et al., 2017). Female sex and APOE ε4 genotype are regarded as the major risk factors for AD. Interestingly, both APOE ε4 genotype and sex profoundly impact metabolism. Chang et al. revealed the sex- and APOE genotype-specific metabolic signatures in AD (Chang et al., 2023). Changes in amino acids were mainly in males, and PCs and tryptophan were in females. The APOE ɛ4 allele specifically affected the metabolism of PCs. In addition, Arnold et al. further confirmed that sex had the potential to modify the association of metabolites with AD-related biomarkers. A significant negative association between the level of palmitoleic acid (C16:1) and glucose uptake measured by FDG-PET was found mainly in the male group (Arnold et al., 2020). Thus, in future metabolomic studies, sex and APOE ɛ4 allele should be considered as confounding factors.

Peripheral metabolic changes may influence central abnormalities through the liver and gut-brain axis. Bile acids (BAs) appear to play a role in AD pathogenesis. When combining the structural MRI, [^18^F] FDG-PET and targeted metabolomics, Nho and colleagues found that bile acids levels were associated with hippocampal volume, cortical thickness and brain glucose metabolism in AD (Nho et al., 2019). For one primary bile acid metabolite, lower cholic acid (CA) levels were associated with decreased hippocampal volume and reduced glucose metabolism. Nevertheless, for bacterially produced conjugated secondary bile acid metabolites, including glycodeoxycholic acid (GDCA), glycolithocholic acid (GLCA), taurodeoxycholic acid (TDCA) and taurolithocholic acid (TLCA), higher levels were associated with decreased hippocampal volume and reduced glucose metabolism.

Currently, there was only one study combining fecal microbiome, targeted serum metabolomics and structural MRI data. Based on a large human cohort, the main finding was that *Odoribacter* was positively associated with hippocampal volume, which might be mediated by acetic acids (Liang et al., 2022). To date, the number of studies for revealing the interactions between gut microbiota, brain and cognition in humans is few. Future studies are needed to elucidate the effect of microbial modulation on cognition and behavior *via* the combination of metabolomics and neuroimaging.

## 5. Discussion and future perspectives

Although accumulating literatures support the contribution of gut microbial dysbiosis to the pathogenesis of AD, the complex interaction mechanisms involving the microbiota-gut-brain axis remain to be fully established. Peripheral metabolites may be a vital intermediate factor in communicating gut microbiota and the brain. Neuroimaging techniques can quantitatively mirror brain structural and functional changes in AD, which can directly reflect the effect of altered gut microbiota and metabolites on the human brain. Currently, most studies have profiled the association of altered microbiome or metabolites with neuroimaging parameters in patients with AD or MCI, whereas few studies focus on their causative correlations and the establishment of diagnostic models using integrated multi-omics techniques, especially in preclinical AD. In the future, the application of integrated neuroimaging-omics analysis has the potential for the identification of early AD. Notably, in the revised NIA-AA diagnostic criteria for AD,^1^ the value of plasma and inflammatory/immune biomarkers are also highlighted. Therefore, the combination of multi-omics indicators with new AD biomarkers may provide more diagnostic information for AD. In addition, longitudinal studies with a larger sample size from multiple centers are also needed, which may reveal the dynamic changes of these multi-omics biomarkers and further validate the robustness of findings. Moreover, the lack of data regarding neuroimaging modalities other than sMRI, DTI, and fMRI also deserves due attention. In conclusion, microbiota-gut-brain interactions may reveal novel insights into the mechanism of AD, and further provide the potential therapeutic target for AD interventions and treatments.

## Author contributions

CS, XW, and SJ conceived the review. CS, WD, and XW drafted the initial manuscript. QD, XC, YL searched literatures. PX and CS revised the manuscript. All authors contributed to the article and approved the submitted version.

## Footnotes

^1^ https://aaic.alz.org/nia-aa.asp
